# Sodium fluoride (NaF) induces the splenic apoptosis via endoplasmic reticulum (ER) stress pathway *in vivo* and *in vitro*

**DOI:** 10.18632/aging.101150

**Published:** 2016-12-27

**Authors:** Huidan Deng, Ping Kuang, Hengmin Cui, Lian Chen, Qin Luo, Jing Fang, Zhicai Zuo, Junliang Deng, Xun Wang, Ling Zhao

**Affiliations:** ^1^ College of Veterinary Medicine, Sichuan Agricultural University, Ya'an 625014, China; ^2^ Key Laboratory of Animal Diseases and Environmental Hazards of Sichuan Province, Ya'an 625014, China

**Keywords:** sodium fluoride (NaF), apoptosis, endoplasmic reticulum(ER) stress, spleen, mouse

## Abstract

At present, there are no reports on the relationship between fluoride-induced apoptosis and endoplasmic reticulum (ER) stress (ER stress) in the spleen of human and animals *in vivo* and *in vitro*. Therefore, the aim of this study was to define sodium fluoride (NaF)-induced apoptosis mediated by ER stress in the spleen of mice *in vivo* and *in vitro*. Apoptosis and expression levels of the ER stress-related proteins were detected by flow cytometry and western blot, respectively. The results showed that NaF treatment increased lymphocytes apoptosis, which was consistent with NaF-caused ER Stress. NaF-caused ER stress was characterized by up-regulating protein expression levels of glucose-regulated protein 78 (BiP) and glucose-regulated protein 94 (GRP94), and by activating unfolded protein response (UPR). The signaling pathway of ER stress-associated apoptosis was activated by up-regulating protein expression levels of cleaved cysteine aspartate specific protease-12 (cleaved caspase-12), growth arrest and DNA damage-inducible gene 153 (Gadd153/CHOP) and phosphorylation of JUN N-terminal kinase (p-JNK). Additionally, our *in vitro* study found that apoptotic rate was decreased with remarkable down-regulation of the cleaved caspase-12, CHOP, p-JNK after ER stress was inhibited by 4-Phenylbutyric acid (4-PBA) treatment. In conclusion, NaF-induced apoptosis may mediated by ER stress in the spleen.

## INTRODUCTION

Fluorine, a gaseous element, reacts with almost all kinds of metal elements to generate different fluorides in nature. Fluoride is an effective caries prophylactic agent, and plays an important role in the deposition of calcium and phosphorus in the bones [1]. However, acute or chronic exposure to fluoride can cause damage to various organs and tissues including the enamel [2], skeletal tissues [3], brain [4], kidney [5, 6], spleen [7], thymus [8], renal toxicity [9], epithelial lung cell [10] and erythrocytes [11, 12].

As a largest peripheral lymphatic organ, spleen contains about one-fourth of the body's lymphocytes and initiates immune responses [13, 14]. The initiation of immune response is charged to the white pulp which surrounds the central arterioles and is densely populated with lymphocytes [14, 15]. Fluoride has been reported to alter the histoarchitecture of spleen by reducing the content of white pulp and increasing lymphocyte infiltration in the red pulp [16]. Das [17] observed disorganization in the spleen of male albino rats after NaF treatment. NaF can also damage human lymphocyte chromosomes and induce adverse effects on the spleen [18].

Apoptosis, or cell suicide, is a self-purification process of programmed cell death that can be activated by a variety of signaling pathways [19]. Intriguingly, recent evidence suggests that ER stress also contribute to apoptosis and that ER stress-induced apoptosis pathways have been a hot research area [20]. The ER, a quality control organelle, is responsible for protein folding, maturation, and trafficking [21, 22]. The abnormalities such as protein misfolding and unfolded protein accumulation in the ER cause ER stress [23-25]. During ER stress, excessive unfolded proteins accumulate in the ER lumen, and then result in the dissociation of Bip from the ER stress transducers, which triggers activation of the UPR branches [26]. ER stress-induced apoptosis can occur when UPR fails to compensate for the abnormalities [27]. Fluoride is reported to induce apoptosis in human thyroid follicular epithelial cells [28] and rat osteoblasts in *vivo* [29] via ER stress pathways.

Fluoride-induced apoptosis is an important mechanism of fluoride cytotoxicity [30] and is known to be associated with the ER stress [31]. Based on the above-mentioned references, there are no reports on the relationship between fluoride-induced apoptosis and ER stress in the spleen of human and animals *in vivo* and *in vitro* at present. Therefore, the *in vitro* and *in vivo* studies were firstly designed to define NaF-induced apoptosis via ER stress pathway in the spleen of mice.

## RESULTS

### Effects of NaF on spleen *in vivo*

#### Effects of NaF on ER stress chaperones in the spleen

Bip and GRP94 are the ERS chaperones. We assessed the protein expression levels of Bip and GRP94 after NaF treatment by western blot.

**Figure 1:**
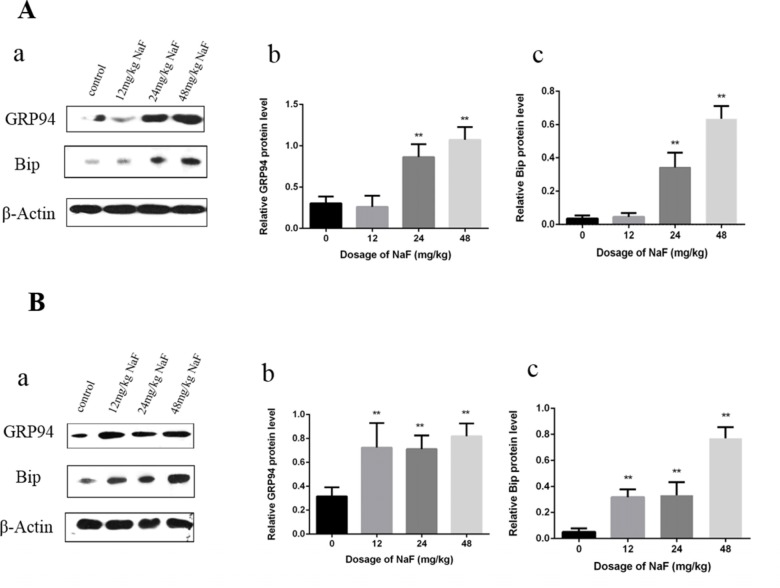
Effect of NaF on ER stress-related proteins in the spleen at 21 (A) and 42 (B) days of age (**a**) The western blot assay. (**b-c**) Quantitative analysis of protein expression. Data are presented with the means ± standard deviation, * *p* < 0.05, ** *p* < 0.01, compared with the control group. Data were analyzed by the variance (ANOVA) test of the SPSS 19.0 software.

At 21 days of age, the results in Figure 1 showed that Bip and GRP94 protein expression levels were significantly increased (P<0.01) in the 24 mg/kg and 48 mg/kg groups in comparison with those in the control group. At 42 days of age, Bip and GRP94 protein expression levels were significantly higher (P<0.01) in the three NaF-treated group than those in the control group.

#### Effects of NaF on UPR in mice spleen

**Figure 2:**
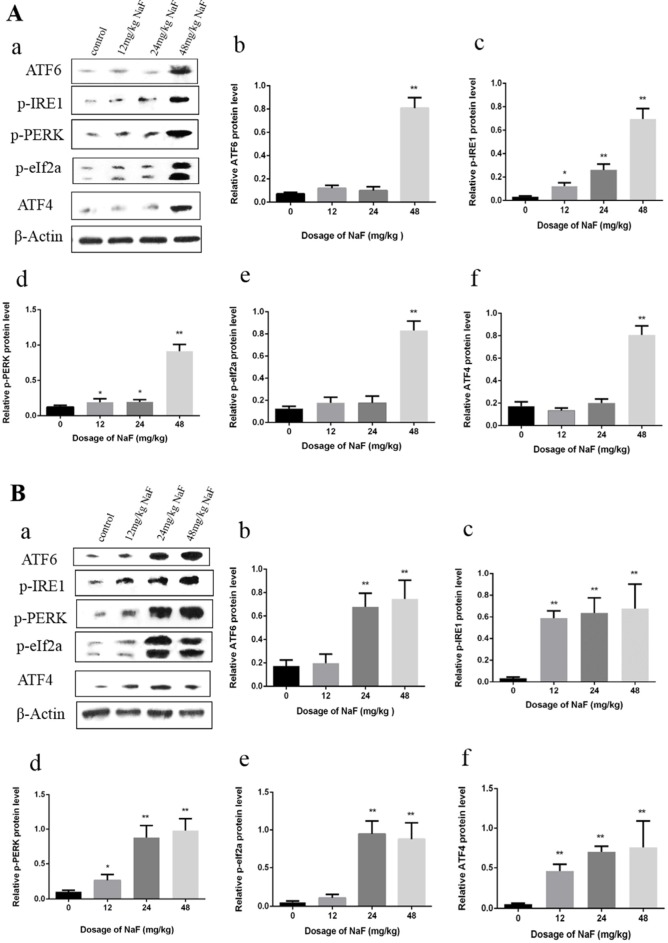
Effect of NaF on protein expression levels of ATF6, p-IRE1, p-PERK, p-eIf2a and ATF4 in the spleen at 21 (A) and 42 (B) days of age (**a)** The western blot assay. (**b-f**) Quantitative analysis of protein expression. Data are presented with the means ± standard deviation, * *p* < 0.05, ** *p* < 0.01, compared with the control group. Data were analyzed by the variance (ANOVA) test of the SPSS 19.0 software.

As shown in Figure 2A, the transcription factor 6 (ATF6), phosphorylation of eukaryotic initiation factor 2α (p-eIF2α) and transcription factor ATF4 protein expression levels were increased (P<0.01) in the 48 mg/kg group at 21 days of age. The phosphorylation of protein kinase RNA (PKR)-like endoplasmic reticulum kinas (p-PERK) and the inositol-requiring enzyme 1 (IRE1) protein expression levels were significantly increased (P<0.01 or P<0.05) in the three NaF-treated groups when compared with those in the control group at 21 days of age.

In the Figure 2B, the ATF6 and p-eIF2a protein expression levels were significantly increased (P<0.01) in the 24 mg/kg and 48 mg/kg groups when compared with those of the control group at 42 days of age. ATF4, p-IRE1, p-PERK protein expression levels were higher (P<0.01 or P<0.05) in the three NaF-treated groups than those in the control group at 42 days of age.

#### Effects of NaF on apoptosis in spleen

**Figure 3:**
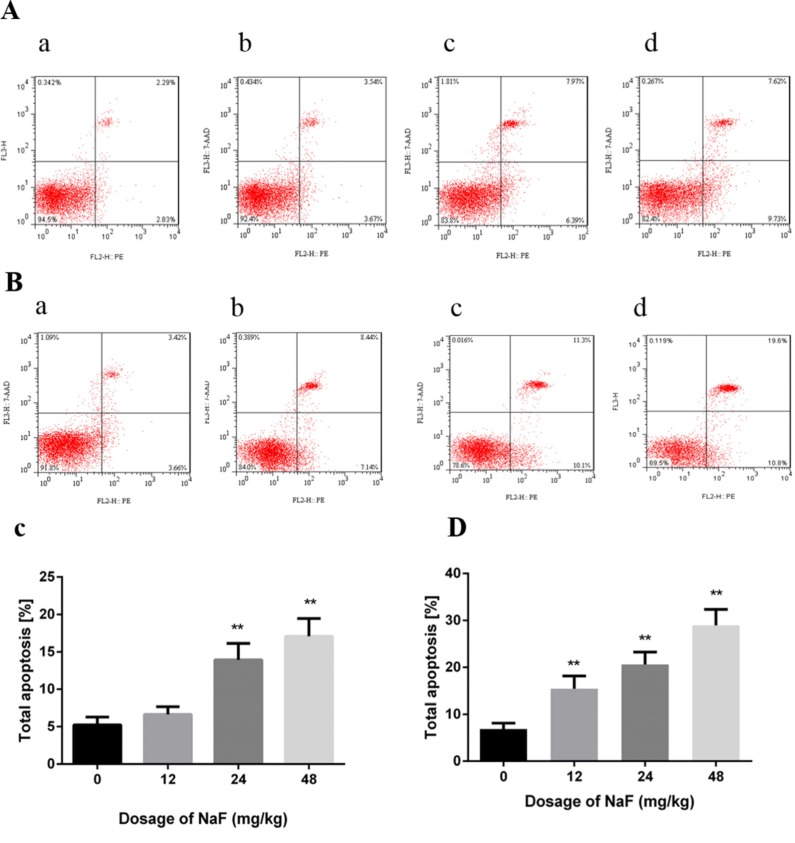
Effect of NaF on apoptosis in the spleen at 21 (A, C) and 42 (B, D) days of age (**a-d**) Two-dimension scatter plots depicting distribution of cells positively stained for Annexin V-PE/7-AAD. (**a**) CG, (**b**) 12 mg/kg group, (**c**) 24 mg/kg group and (**d**) 48 mg/kg group. (**C-D**) Quantitative analysis of total apoptotic lymphocytes. Data are presented with the means ± standard deviation, * *p* < 0.05, ** *p* < 0.01, compared with the control group. Data were analyzed by the variance (ANOVA) test of the SPSS 19.0 software.

The flow cytometry assay showed that apoptotic cells were significantly higher (P<0.01) in the 24 mg/kg and 48 mg/kg groups than those in the control group at 21 days of age. Meanwhile, apoptotic splenocytes were dramatically increased (P<0.01) in the three NaF-treated groups when compared with those of the control group at 42 days of age. The results were shown in Figure 3.

#### Effects of NaF on proteins of ER stress-associated spoptosis in the spleen

**Figure 4:**
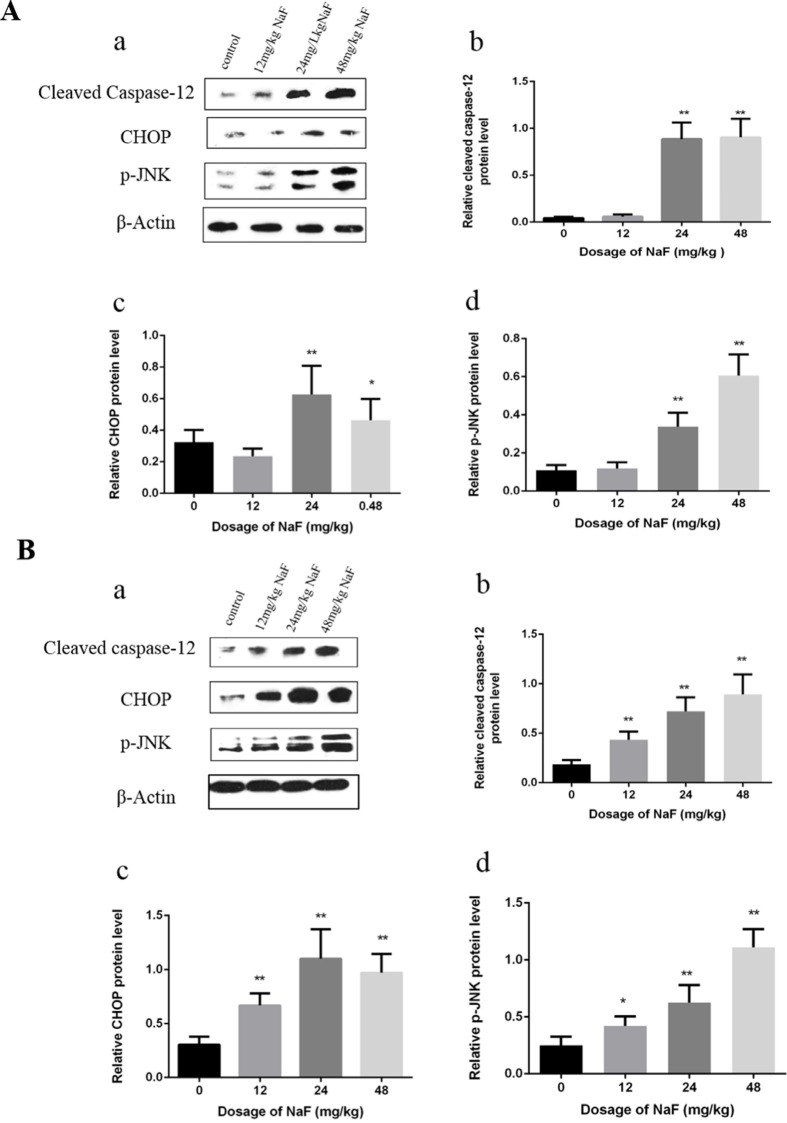
Effect of NaF on apoptosis-related protein in mice spleen at 21(A) and 42 (B) days of age (**a**) The western blot assay. (**b-d**) Quantitative analysis of protein expression. Data are presented with the means ± standard deviation, * *p* < 0.05, ** *p* < 0.01, compared with the control group. Data were analyzed by the variance (ANOVA) test of the SPSS 19.0 software.

Western blot analysis demonstrated that the protein levels of cleaved caspase-12, p-JNK and CHOP were significantly increased (P<0.01 or P<0.05) in the 24 mg/kg and 48 mg/kg groups in comparison with those in the control group at 21 days of age. Furthermore, protein expression levels of cleaved caspase-12, p-JNK and CHOP were higher (P<0.01 or P<0.05) in the three NaF-treated groups than those in the control group at 42 days of age. The results were shown in Figure 4.

### Effects of NaF on splenic lymphocytes of mice *in vitro*

In order to support the above-mentioned *in vivo* findings, we also investigated whether NaF could induce ER stress and apoptosis *in vitro*.

#### Effects of NaF on ER stress chaperones in splenic lymphocytes

**Figure 5:**
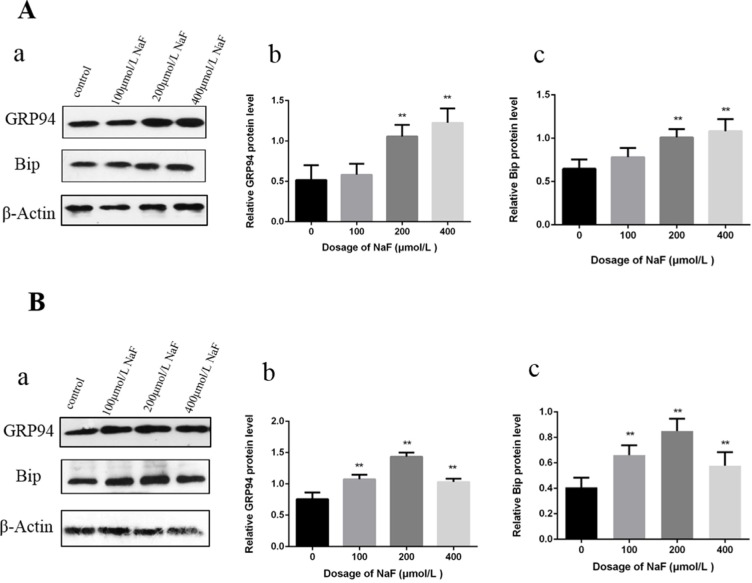
Effect of NaF on protein expression levels of GRP94 and Bip in cultured splenic lymphocytes at 24 h (A) and 48 h (B) (**a**) The western blot assay. (**b**-**c**) Quantitative analysis of protein expression. Data are presented with the means ± standard deviation, * p < 0.05, ** p < 0.01, compared with the control group. Data were analyzed by the variance (ANOVA) test of the SPSS 19.0 software.

As shown in Figure 5, Bip and GRP 94 protein expression levels were significantly higher (P<0.01) in the MG and HG than those in the control group after NaF treatment for 24 h. Then, Bip and GRP94 protein expression levels were significantly increased (P<0.01) in the LG, MG and HG, and peaked in the MG when compared with those in the CG after NaF treatment for 48 h.

#### Effects of NaF on UPR in splenic lymphocytes

To investigate whether the NaF activated UPR, western blot was used to detect the protein expression levels of p-IRE1, p-PERK, ATF6, p-eIF2a and ATF4.

**Figure 6:**
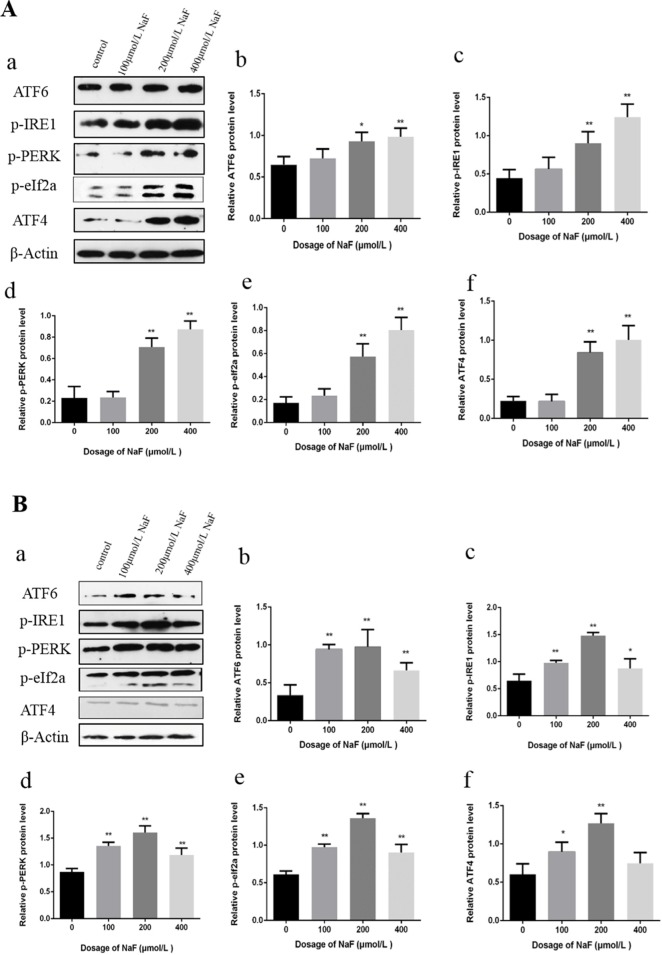
Effect of NaF on protein expression levels of ATF6, p-IRE1, p-PERK, p-eIf2a and ATF4 in cultured splenic lymphocytes at 24 h (A) and 48 h (B) (**a**) The western blot assay. (**b-f**) Quantitative analysis of protein expression. Data are presented with the means ± standard deviation, * *p* < 0.05, ** *p* < 0.01, compared with the control group. Data were analyzed by the variance (ANOVA) test of the SPSS 19.0 software.

In Figure 6, p-IRE1, p-PERK, ATF6, p-JNK, p-eIF2a and ATF4 protein expression levels were significantly higher (P<0.01 or P<0.05) in the MG and HG than those in the CG after NaF treatment for 24 h. Moreover, NaF treatment for 48 h significantly increased (P<0.01 or P<0.05) p-IRE1, p-PERK, ATF6 and p-eIF2a protein expression levels in the LG, MG and HG, and peaked in the MG when compared to CG. ATF4 protein expression levels were increased (P<0.01 or P<0.05) in the LG and MG in comparison with those of the CG.

#### Effects of NaF on apoptosis in splenic lymphocytes

Our previous study have proved that NaF can induce apoptosis in splenic lymphcytes [32]. The results showed that apoptotic lymphocytes were significantly higher (P<0.01) in the MG and HG than those in the LG and CG after NaF treatment for 24 h. There was no significantly difference between LG and CG. Meanwhile, after NaF treatment for 48 h, apoptotic lymphocytes were dramatically increased (P<0.01) among NaF-treated groups and control group and peaked in the MG.

#### Effects of NaF on proteins of ER stress-associated apoptosis in splenic lymphocytes

**Figure 7:**
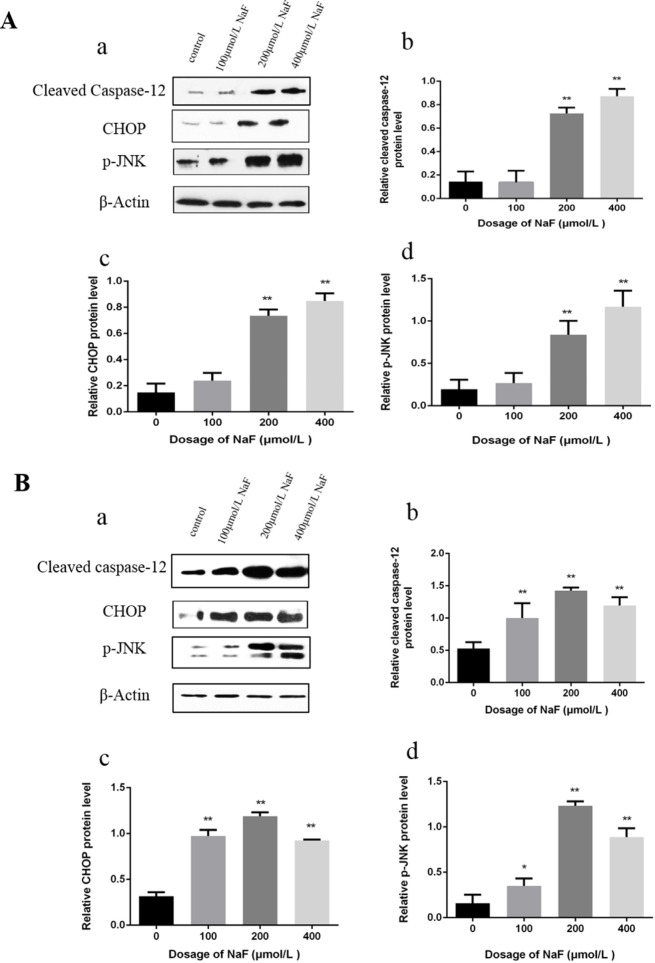
Effect of NaF treatment on protein expression levels of cleaved caspase-12, p-JNK, CHOP in cultured splenic lymphocytes at 24 h (A) and 48 h (B) (**a**) The western blot assay. (**b-d**) Quantitative analysis of protein expression. Data are presented with the means ± standard deviation, * *p* < 0.05, ** *p* < 0.01, compared with the control group. Data were analyzed by the variance (ANOVA) test of the SPSS 19.0 software.

Western blot analysis demonstrated that the protein level of cleaved caspase-12, p-JNK and CHOP were significantly increased (P<0.01) in the MG and HG in comparison with those in the control group after NaF treatment for 24 h. Moreover, the protein expression levels of cleaved caspase-12, p-JNK and CHOP were higher (P<0.01 or P<0.05) in the LG, MG and HG than those in the CG exposure to NaF for 48 h. The results were shown in Figure 7.

#### Effect of ER stress inhibitor 4-PBA on splenic lymphocytes viability

**Figure 8:**
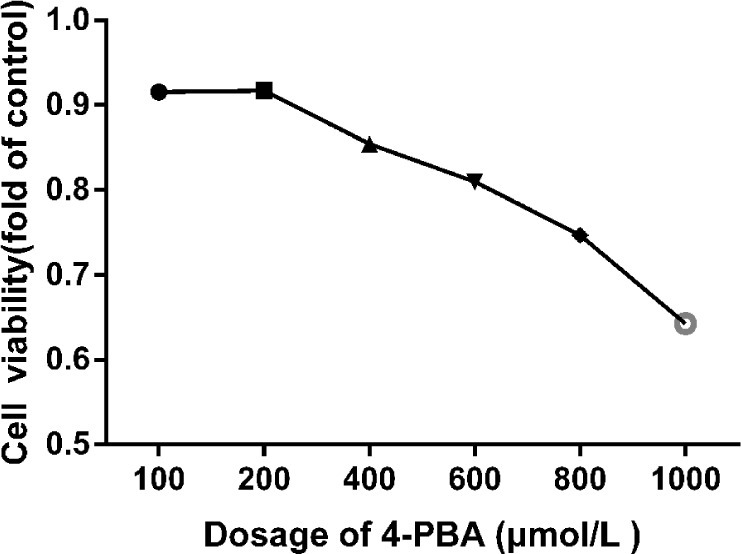
Cell viabilities of 4-PBA treated splenic lymphocytes for 48 h The cell viability was determined using a CCK8 assay as described in the methodology section. The splenic lymphocytes were incubated with different 4-PBA concentrations for 48 h. Data were analyzed by the variance (ANOVA) test of the SPSS 19.0 software.

CCK-8 assay was performed to test the cytotoxic effect of 4-PBA on splenic lymphocytes viability. As shown in Figure 8, viability of the splenic lymphocytes was significantly decreased (P < 0.05) at 400 μmol/L of NaF exposure for 48 h. Approximate 90% of cells were survived at 200 μmol/L 4-PBA. Based on these findings, treatment of 200 μmol/L 4-PBA was selected for further study.

#### Effect of NaF-combined with ER stress inhibitor 4-PBA on ER Stress Chaperones in splenic lymphocytes

**Figure 9:**
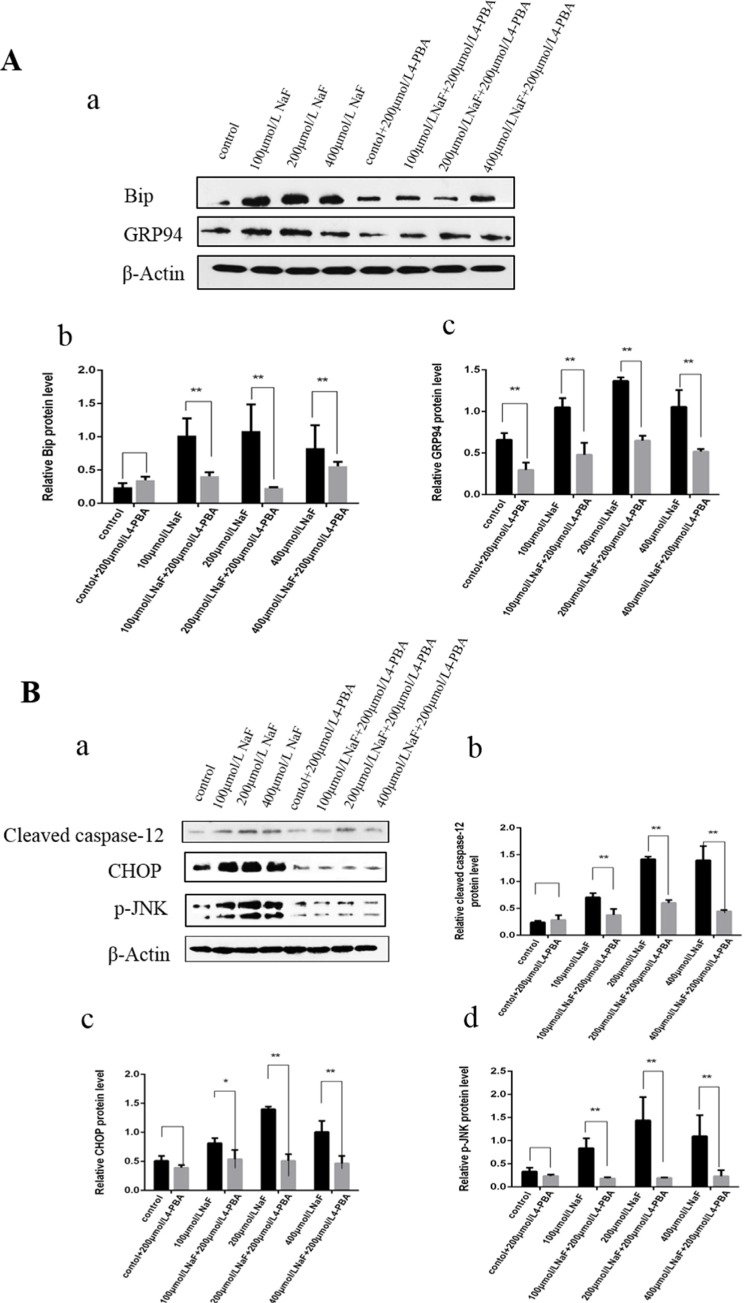
Effect of NaF and NaF-combined with 4-PBA treatment on protein expression levels of Bip, GRP94, cleaved caspase-12, p-JNK and CHOP in cultured splenic lymphocytes at 48 h (**a**) The western blot assay. (**b-c**) Quantitative analysis of protein expression. Data are presented with the means ± standard deviation, * *p* < 0.05, ** *p* < 0.01, compared with the control group. Data were analyzed by the variance (ANOVA) test of the SPSS 19.0 software.

Protein folding in the ER is facilitated by ER chaperone proteins such as Bip/GRP78 and GRP94. These proteins are induced by ER stress [33]. To investigate whether 4-PBA inhibited the ER Stress, western blot was used to detect the protein expression of ER stress markers Bip and GRP94 after treatment with 4-PBA and/or NaF for 48 h. As shown in Figure 9A, the expressions of Bip and GRP94 protein significantly decreased (P<0.01) in the NaF-combined 4-PBA groups when compared with those of the NaF groups, which indicated that 4-PBA inhibited ER Stress effectively.

#### Effect of NaF-combined with ER stress inhibitor 4-PBA on proteins of ER stress-associated apoptosis in splenic lymphocytes

Results of western blot analysis (Figure 9B) showed that the protein expression levels of cleaved caspase-12, p-JNK and CHOP were significantly decreased (P<0.01 or P<0.05) in the NaF-combined 4-PBA groups when compared with those of the NaF groups. Collectively, the suppression of ER stress by 4-PBA-combined with NaF decreased apoptosis by down-regulating protein expression levels of cleaved caspase-12, p-JNK and CHOP in splenic lymphocytes.

#### Effect of NaF-combined with ER stress inhibitor 4-PBA on apoptosis in splenic lymphocytes

**Figure 10:**
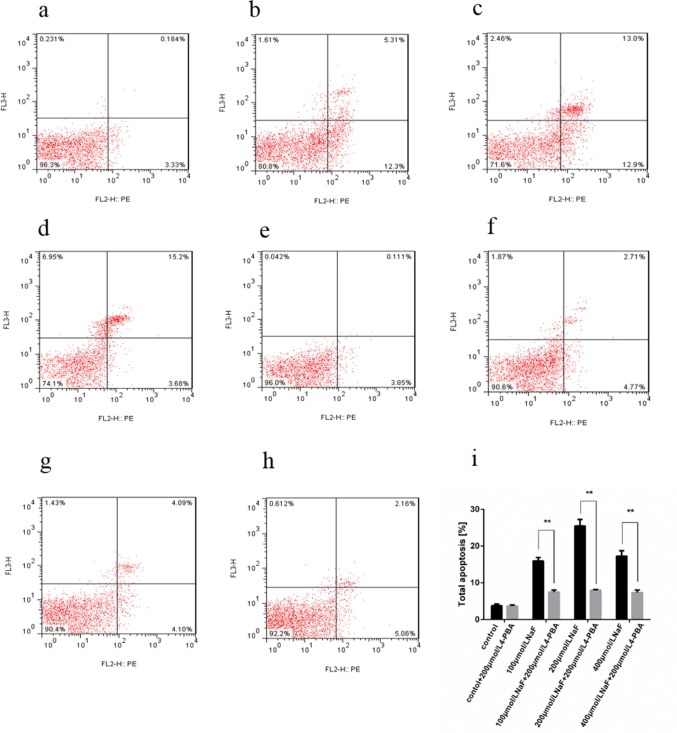
Effect of NaF and NaF-combined with 200 μmol/L 4-PBA treatment on apoptosis of cultured splenic lymphocytes at 48 h (**a-h**) Two-dimension scatter plots depicting distribution of cells positively stained for Annexin V-PE/7-AAD. (**a**) CG, (**b**) LG, (**c**) MG and (**d**) HG, (**e**) CG+4-PBA, (**f**) LG+4-PBA, (**g**) MG+4-PBA, (**h**) HG+4-PBA. (**i**) Quantitative analysis of total apoptotic lymphocytes. Data are presented with the means ± standard deviation, * *p* < 0.05, ** *p* < 0.01, compared with the control group. Data were analyzed by the variance (ANOVA) test of the SPSS 19.0 software.

Cells in lower left quadrant of each picture correspond to normal cells. Cells in right lower quadrant correspond to early apoptotic cells. Cells in right upper quadrant correspond to late apoptotic. Cells in left upper quadrant correspond to dead cells. With or without 4-PBA, cells were treated with NaF for 48 h. Then, the apoptotic rates were determined by flow cytometry with double staining of Annexin V-PE/7-AAD. The total apoptotic rate was significantly decreased (P<0.01) in the both 4-PBA and NaF-treated groups than those in the only NaF-treated groups for 48 h, as shown in Figure 10.

## DISCUSSION

Indeed, our *in vivo* and *in vitro* studies found that NaF could induce apoptosis and ER stress. The NaF-caused ER stress was characterized by up-regulating protein expression levels of BiP and GRP94, and by activating UPR. Concurrently, the studies firstly proved the NaF-induced ER stress-associated apoptotic pathway in the spleen.

Proper function of the ER is essential to cell survival and any perturbation of its function induces cellular damage and results in apoptosis. Various conditions can disturb ER functions, events collectively termed “ER stress” [33]. Resident chaperones like BiP and GRP94 in the ER lumen facilitate the proper folding of the nascent protein [34] and these chaperones are induced by ER stress. Under ER stress conditions, cells activate an intracellular signaling pathway, known as UPR by activating ER stress transducer proteins such as PERK [35], IRE1[36] and ATF6 [37]. The activation of PERK increases eIF2*α* phosphorylation, and then causes a global attenuation of protein synthesis and a concomitant increase in ATF4 translation [38]. It has been reported that fluoride induces Bip protein expression levels [29], and that fluoride increases mRNA expression levels of Bip and PERK on bone of rats and MC3T3-E1 cells *in vitro* [39]. Our data indicated that NaF increased the protein expression levels of Bip, GRP94, P-IRE1, P-PERK, ATF6, P-eIF2a and ATF4 *in vivo* and *in vitro*, which demonstrated that NaF induced the ER stress in the spleen *in vivo* and *in vitro*.

Severe and prolonged ER stress results in cell death via apoptosis. Several apoptosis pathways, triggered by ER stress, have been reported [40]. The first apoptotic pathway in ER stress is the activation of CHOP. CHOP is a major stress-inducible proapoptotic gene in ER stress-induced apoptosis. All three branches of the UPR regulate the activation of CHOP. CHOP is expressed at a very low level under physiological conditions, but its expression level significantly increases in the presence of severe or persistent ER stress. Overexpression of CHOP leads to apoptosis [41].

The second apoptotic pathway in ER stress is activation of the JNK pathway [37]. ER stress activates JNKs through activation of IRE1α or IRE1β [42]. The IRE1s recruits and activates TRAF2, which further activates JNK [43], and sustained activation of JNK leads cells to apoptosis [44].

The third apoptotic pathway in ER stress is activation of caspase-12 [45]. Caspase-12, as a member of the inflammatory group of the caspase family, localizes in the ER. Moreover, it is specifically activated by ER stress [45]. During the ER stress, the cytosolic domain of IRE1 recruits tumor necrosis factor (TNF) receptor-associated factor 2 (TRAF2), which interacts with caspase-12 and induces the cleavage and activation of the caspase-12 [46]. In turn, activated caspase-12 results in apoptosis [47].

In the present study, the flow cytometry and western blot results showed that NaF induced apoptosis and increased the protein expression levels of CHOP, cleaved-caspase12 and p-JNK *in vivo* and *in vitro*. To further clarify whether ER stress plays an important role in the apoptosis, we used flow cytometry and western blot to observe the changes of apoptosis after ER stress inhibitor 4-PBA and NaF treatment *in vitro*. The western blot results showed decreased protein expression levels of Bip, GRP94, CHOP, cleaved-caspase12 and p-JNK. In addition, the apoptosis rate measured by flow cytometry was markedly decreased in which ER stress had been inhibited. These results suggest that the NaF induced apoptosis via ER stress pathway in the spleen of mice.

### Conclusions

Based on the results of our *in vivo* and *in vitro* studies, it is concluded that NaF induces apoptosis and ER stress, and that NaF-caused ER stress pathway may mediate apoptosis by up-regulating protein expression levels of cleaved caspase-12, CHOP, p-JNK in the spleen. The up-regulation of protein expression levels of BiP and GRP94, and activation of UPR are molecular basis of NaF-caused ER stress.

## MATERIALS AND METHODS

### Chemicals and supplies

NaF (S6776) was purchased from Sigma Aldrich, UK. Lymphocyte separation medium (DKW33-R0100) were supplied by Dakewe Biotech Company, China. RIPA lysis buffer (P0013C), BCA Protein Assay Kit (P0012), Cell Counting Kit-8 (CCK-8) (C0038) and Annexin V-PE/7-AAD Apoptosis Detection Kit I (559763) were obtained from BD, USA. RPMI 1640 (11875119) and fetal bovine serum (16000044) were supplied by Gibco, UK. The mouse Bip (3177T), GRP94 (20292T), CHOP (2895P), ATF6 (65880S), p-JNK (4668T), p-eIF2a (3398P), ATF4 (11815S), cleaved caspase-12 (ab18766) antibodies, mouse IgG (7076P2) and rabbit IgG (7074P2) were obtained from Cell Signaling Technology, UK. p-PERK (sc-32577) were obtained from Santa Cruz Biotechnology, USA, p-IRE1 (ab48187) were obtained from Abcam, UK.

### Lymphocyte isolation, culture and treatment

The ICR mice were obtained from the Experimental Animal Corporation of DOSSY Biological Technology Company. 3-week-old ICR mice were anaesthetized and euthanized. After laparotomy, the spleen was separated from the mice and washed with cold phosphate buffered saline (PBS, pH 7.4). Then, the spleen was placed in a 200-mesh stain steel sieve over a culture dish containing 4-5 mL lymphocyte isolation separation medium and grounded into small pieces with the plunger of glass syringe. The liquid was transferred into a centrifuge tube, and then 200-500 μl RPMI–1640 medium was added and centrifuged at 800×*g* for 30 min at room temperature. Three layers were formed after centrifugation. The middle milky layer which contained lymphocytes was transferred into a test tube. The lymphocytes were washed twice with PBS and suspended in RPMI-1640 medium with 10 % fetal calf serum and then transferred into a culture bottle. All these processes were performed under sterile condition. The viability of the lymphocyte was estimated according to the trypan blue exclusion criteria, and the viability is over 95%.

To monitor various parameters (except the CCK-8 bioas-say), splenic lymphocytes were cultured in the RPMI-1640 medium (supplemented with 10% fetal calf serum, 100 U/mL penicillin, 100 μg/mL streptomycin) containing 0 (control group, CG), 100(low-dose group, LG), 200 (medial-dose group, MG), and 400 (high-dose group, HG) μmol/L NaF. There were three repeats in the each treatment. All cells were maintained in a humidified incubator for 24 and 48h at 37°C with 5% CO2.

### Animals and treatment

240 healthy ICR mice (Experimental Animal Corporation of DOSSY at Chengdu, China) were used in this experiment. Food and water was provided ad libitum. The mice were divided into 4 groups (N=60). The control group was given an intragastric administration of distilled water at the same time as other groups. The experimental groups were given an intragastric administration of 12, 24, and 48 mg/kg NaF (ChengDu Kelong Chemical Co., Ltd., Chengdu, China), respectively. NaF was diluted by distilled water. The gavage doses of four groups were 1 ml/100g animal weight once daily for 42 days. At 21 and 42 days of age, the spleen samples were taken from mice. There were eight repeats in the each treatment.

Our experiments involving the use of mice and all experimental procedures were approved by the Animal Care and Use Committee, Sichuan Agricultural University.

### Flow cytometry analysis

The percentage of apoptosis was evaluated using an Annexin V-PE/7-AAD detection kit (BD Biosciences, San Jose, CA, USA) according to the manufacturer's protocol.

*In vitro* experiment, cells were cultured in the presence of 0, 100, 200 and 400 μmol/l NaF for 24 and 48 h. Prior to toxicity detection, the cells were collected and washed two times with phosphate buffered saline (PBS, PH 7.4). Pellets were collected and resuspend in 100 μl PBS, and stained with PE Annexin V and 7-amino-actinomycin (7-AAD) for 15 minutes in the dark. Then, 400 μL binding buffer (BD Pharmingen) was added. Data were then obtained by a FACSCalibur (Becton Dickinson, USA).

*In vivo* experiment, mice were humanely killed at 21 and 42 days of age, spleens were taken from each mouse and ground to form a cell suspension that was filtered through a 300-mesh nylon screen. The cells were washed twice with cold PBS (phosphate buffer solution, pH 7.2-7.4) and then suspended in PBS at a concentration of 1×10^6^ cells/mL. 100 μL portions of the cell suspension were transferred into 5 mL culture tubes, and stained with PE Annexin V and 7-amino-actinomycin (7-AAD). The mixture was gently vortexed and incubated for 15 min in the dark. 400 μL of 1×binding buffer was added to each tube, and analysis by FACSCalibur (BD FACSCalibur).

### Western blot analysis

Cells and spleen tissues were lysed and proteins were extracted with RIPA lysis buffer and then kept in laemmli buffer. Protein samples were resolved on SDS-PAGE (10%–15% gels) and transferred to nitrocellulose filter membranes. The membranes were blocked in 5% nonfat dry milk for 1 h and then incubated with the primary antibodies overnight at 4°. The primary antibodies were Bip, Grp94, CHOP, ATF6, P-PERK, P-IRE1, P-JNK, P-eIF2a, ATF4 and cleaved caspase-12. The membranes were then washed with PBS-Tween (PBST) and incubated with the biotin-conjugated secondary antibodies for 1 h, and washed again with PBS-Tween (PBST). Blots were visualized by ECL^TM^ (Bio-Rad) and X-ray film. When the strip is thicker and darker, protein expression is higher, otherwise it lower. Then, the statistical data of protein expression was done with ImageJ2x software.

### Measurement of cell viability

Cell viability was assessed using the CCK-8 bioassay[48, 49]. Splenic lymphocytes (7×105/well) were seeded into 96-well flat-bottomed plates and were exposed to 4-PBA(0–1000 μmol/L) for 48 h at 37° with 5% CO2. After NaF exposure, 10 μL CCK-8 solutions were added to each well, and incubated for 4 h. The optical density (OD) was measured at 450 nm using a microplate reader.

### Statistical analysis

All the data were analyzed by SPSS 19.0. All the results were expressed as mean±SD. Data were analyzed by one way analysis of variance (ANOVA). P<0.05 was considered as a significant difference.
